# Recent Advances in Sustainable Management of *Cylas formicarius*

**DOI:** 10.3390/insects17030245

**Published:** 2026-02-26

**Authors:** Youmiao Li, Henan Ju, Wanqiu Huang, Baolin Ou, Huifeng Li, Yongmei Huang, Yanqing Li, Tianyuan Chen, Xia-Lin Zheng, Jinfeng Hua

**Affiliations:** 1 Department of Sweet Potato Genetic Breeding and Application, Institute of Maize Research, Guangxi Academy of Agricultural Sciences, Nanning 530007, China; 19978672869@163.com (Y.L.); juhenan123@163.com (H.J.); wanqiu8311@163.com (W.H.); oubaolin1203@163.com (B.O.); 605lifeng@163.com (H.L.); huangyongmei322@163.com (Y.H.); liyq2004.7@163.com (Y.L.); tianyuanchen@126.com (T.C.); 2 College of Agriculture, Guangxi University, Nanning 530004, China

**Keywords:** *Cylas formicarius*, biological control, entomopathogens, natural enemies, botanical pesticides, sex pheromone

## Abstract

*Cylas formicarius* is a significant quarantine pest worldwide, causing year-round outbreaks in tropical and subtropical regions. This pest is characterized by a highly cryptic nature, a high reproduction rate, and broad environmental adaptability, which makes it difficult to control. Frequent use of pesticides is not cost-effective, as it brings the accompanying effects of pesticide residues and environmental pollution. With the rapid development of green agriculture, biological control has become an important component in an integrated green management system for *C. formicarius.* Therefore, this paper critically reviews the research progress in insect pathogenic microorganisms, plant-based pesticides, natural enemy insects, insect sex pheromones, transgenic technology, as well as the screening, identification, and breeding of insect-resistant varieties. The aim is to provide theoretical references for the research and application of sustainable management of *C. formicarius.*

## Simple Summary

This review summarizes advances in the biological control of *Cylas formicarius*, a major quarantine pest of sweet potatoes. Traditional chemical control methods face challenges such as resistance and environmental hazards, creating an urgent need for green, safe, and sustainable control methods for prevention and management of *Cylas formicarius.* The paper reviews several promising biocontrol strategies: entomopathogenic fungi (e.g., *Beauveria bassiana*, *Metarhizium anisopliae*) and nematodes show strong virulence; botanical pesticides like azadirachtin offer low-toxicity options; sex pheromone traps are effective for monitoring and mass trapping; and transgenic technology along with RNA interference (RNAi) provide novel genetic approaches. Additionally, breeding resistant sweet potato varieties is highlighted as a sustainable long-term solution. The review concludes by identifying current challenges and future research directions to establish an integrated green management system for this pervasive pest.

## 1. Introduction

Sweet potatoes (*Ipomoea batatas* (L.) Lam) play a crucial role in ensuring global food security and increasing farmers’ income as an important food crop, feed source, industrial raw material, and energy crop [1,2,3]. According to statistics from the Food and Agriculture Organization of the United Nations (FAO), the global sweet potato planting area reached 7.570 × 10^6^ hm^2^ in 2023, with a total output of approximately 9.352 × 10^7^ t. The main production areas are located in China, Malawi, Nigeria, Tanzania, Uganda, and Indonesia [4]. With the rapid development of the sweet potato industry, its market demand continues to grow. However, the increasing occurrence and expanding damage scope of *Cylas formicarius* Fabricius (Coleoptera: Brentidae), which is due to global warming, agricultural migration, and the deepening of international trade, pose a severe challenge to the sweet potato industry [5,6].

Sweet potato weevils, *C. formicarius*, are a quarantine pest worldwide [7,8]. The larvae feed within the tuber, forming tunnels that lead the tuber to emit a foul odor, rot, and deteriorate, thus losing its edible value. Meanwhile, the adults primarily feed on the stems, leaves, and young shoots of the tubers and bore into the tubers, seriously affecting the growth of the tubers and readily causing diseases [9,10,11]. According to available statistics, sweet potatoes are cultivated in 109 countries worldwide [12]. More than 80 of these countries have sweet potatoes that are affected by *C. formicarius* [13], which can result in yield losses of 30% to 100%. Moreover, the extent of the damage continues to expand, which is a serious threat to the sustainability of the sweet potato industry [8,14,15,16].

Currently, chemical control remains the main approach to control *C. formicarius* [17,18]. Common chemical insecticides used to control *C. formicarius* mainly include cyantraniliprole, bifenthrin, carbofuran granules and clothianidin [19,20,21]. Once *C. formicarius* invades the tuber crowns or storage roots, chemical control methods (such as toxic baits, dusting, spraying, fumigation, or applying granular agents in furrows) have limited effectiveness. Moreover, due to the migratory behavior of the adults, frequent applications are required to kill adults that migrate from other areas. In developing countries, frequent insecticide use not only incurs high costs but also leads to issues of pesticide residues and environmental pollution [22,23,24,25,26]. Therefore, the development of eco-friendly biological and integrated pest management strategies has become an inevitable trend for controlling *C. formicarius*, representing a key to ensuring the sustainable development of the global sweet potato industry [27].

## 2. Life Cycle and Damage

**Figure 1:**
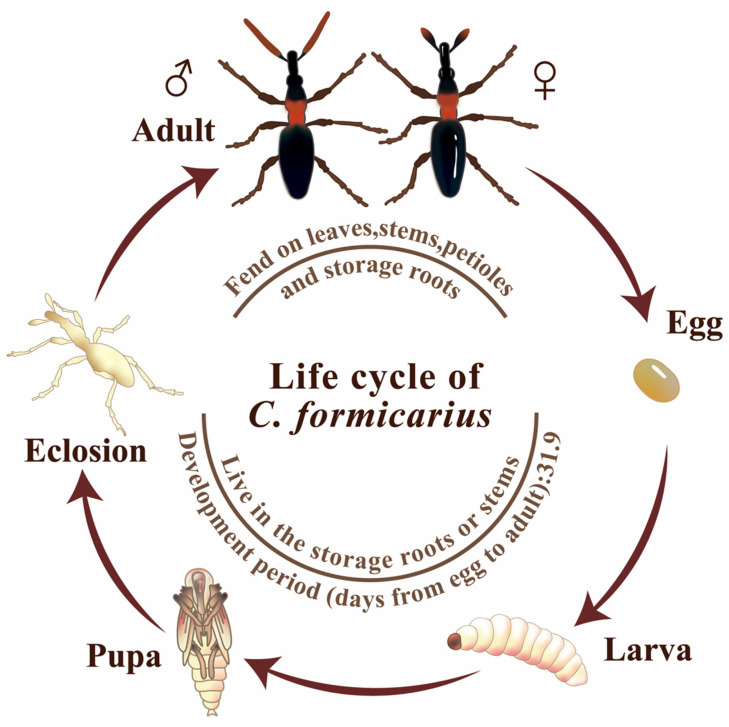
Life cycle of *C. formicarius*.

The life cycle of *C. formicarius* comprises four developmental stages: egg, larva, pupa, and adult [11,14]. Eggs are oval-shaped, measuring 0.62–0.70 mm in length and 0.43–0.46 mm in width. Upon oviposition, they appear milky white and transition to pale yellow prior to hatching. The larvae are cylindrical, the thorax and abdomen are milky white in coloration, and the “grubs” are legless with brown heads. Pupae are initially white and gradually change from pale yellow to dark brown as development proceeds. Adults exhibit a sclerotized body wall; the antennae, prothorax, and legs are orange-red to reddish-brown, while the remaining body parts are blue-black with a metallic sheen. The head is extended anteriorly into a pronounced rostrum resembling an elephant’s trunk, with chewing mouthparts situated at its apex. Antennal morphology is sexually dimorphic: males have filiform antennae, whereas females exhibit clavate antennae (Figure 1) [9,10,11,14,26]. Under optimal temperatures of 27–30 °C, the complete life cycle lasts approximately 31.9 days, and adult longevity can reach 93–95 days [14,26].

The adults feed on leaves, petioles, and stems, resulting in oval-shaped feeding holes; severe infestations may induce leaf wilting [10,11,14]. Adults also create circular feeding punctures on tuber surfaces. Females preferentially oviposit in tubers, depositing eggs in shallow cavities 1–4 mm deep [14]. These oviposition sites are sealed with grayish frass, which serves to maintain humidity, conceal the eggs, and reduce predation risk. Larvae and pupae feed internally within vines and tubers, causing tissue swelling and cracking. Furthermore, larval feeding induces the synthesis of terpenoid phytoalexins in tubers, rendering infested portions unpalatable [11,14]. Overall, heavy infestation leads to leaf chlorosis, reduced plant vigor, and impaired growth. Vine damage is positively correlated with tuber damage, ultimately resulting in yield reduction and the production of smaller tubers [14,15,16].

## 3. Insect Pathogenic Microorganisms

Insect-pathogenic microorganisms denote those microorganisms capable of infecting insects, inducing disease in them, and causing disease prevalence within insect populations. They represent a crucial category of natural factors that regulate the dynamics of insect population size [28].

### 3.1. Entomopathogenic Fungi

Entomopathogenic fungi (EPF) are the most abundant group of entomopathogenic organisms and can effectively control the occurrence of *C. formicarius* [29]. Fungal insecticides primarily facilitate the attachment of fungal conidia to the insect cuticle, where the conidia subsequently absorb moisture, germinate, and develop into germ tubes or form appressoria. These structures then penetrate the insect body, inducing pathological alterations and physical impairment that ultimately result in host mortality [16,30]. To date, the most widely utilized entomopathogenic fungi for the biological suppression of *C. formicarius* include *Beauveria bassiana* and *Metarhizium anisopliae* [15,16,29] (Table 1).

Extensive empirical studies have consistently demonstrated that *B. bassiana* and *M. anisopliae* exhibit potent biocontrol efficacy against *C. formicarius*. For example, *B. bassiana* strains isolated from soils have been documented to induce a cumulative mortality rate in *C. formicarius* populations exceeding 80% [31]. Ondiaka et al. found that *B. bassiana* had a mortality rate of 77.5% to 84.2% for *C. formicarius*; spraying with 1.0 × 10^8^ *B. bassiana* suspension, the weight of the tubers consumed by the treatment group (6.9 g) was 8.7 g less than that of the control group (15.6 g) [32]. Cao et al. reported *B. bassiana* strains Gxj-10 and Xwy-1 have 15-day corrected adult mortality rates of 82.1% and 60.7% respectively, and they had high spore production characteristics suitable for industrial production [33]. In addition, Prayogo et al. compared the field control effects of *B. bassiana* with the chemical pesticide deltamethrin and found that *B. bassiana* not only had better control effects but also reduced root yield loss by 19% [29].

As regards *M. anisopliae*, Ondiaka et al. further investigated the virulence of *M. anisopliae* and its effects on the reproductive capacity of *C. formicarius* [32]. The results showed that the mortality rate of *C. formicarius* in the treatment group was 62.5–89.2%, after spraying with 1.0 × 10^8^ *M. anisopliae* suspension, while the food intake was reduced by 10.4 g compared to the control groups (15.6 g). When female weevils were treated with a 3.0 × 10^7^ *M. anisopliae* suspension, their egg production (4.7 eggs per female) was significantly lower than that of the control groups (16.3 eggs per female). The *M. anisopliae* complex strains from different hosts were all pathogenic to *C. formicarius*, among which the LBM-30 strain had a 7-day cumulative mortality rate of up to 96.6% at a concentration of 5 × 10^7^ conidia/mL, metabolites such as amylase, protease, caseinase, chitinase and lipase produced by the strain were closely associated with pathogenicity [34]. Another study, paired feeding and other experiments, evaluated the effects of *M. anisopliae* strain QS155 (1.5 × 10^6^ conidia/mL) on the reproduction and spread of *C. formicarius.* It was found that in the treatment group, female egg production decreased significantly by 51.3–67.8%, egg-laying behavior was abnormal (more eggs exposed outside the sweet potato tubers), and egg hatching rate was reduced. Overall reproductive capacity decreased by 69% to 80%. The strain can also cause a high mortality rate of 63% through horizontal transmission, showing potential for sustained population control [35].

A virulence assay of six Cuban-derived *M. anisopliae* showed that strain LBM-267 had a 100% mortality rate within 7 days at a concentration of 5 × 10^7^ spores/mL, with an LC_50_ of 2.7 × 10^6^ spores/mL and strong spore production capacity, indicating development potential [16]. Another study found that oral ingestion of the *M. anisopliae* strain MR had the highest mortality rate of 62.47% in *C. formicarius* [36]. Hawaiian origin *M. anisopliae* Ko-002, after composting improvement application, reduced the population density of *C. formicarius* by 84% and cut yield loss by 50% [37]. In addition to *B. bassiana* and *M. anisopliae*, other fungi such as *Lecanicillium lecanii* also showed significant control effects, with a mortality rate of 74% at 1 × 10^9^ spores/mL [38]. In summary, entomopathogenic fungi are known to be pathogenic to *C. formicarius*, but the control effects of different strains vary significantly [16,19,32,39]. In the future, research on the biological and molecular characteristics of highly effective strains should be expanded to provide a basis for their large-scale production and application.

**Table 1:** Reported efficacy of various fungal species in controlling *C. formicarius*.

Fungal Species	Experimental Conditions	Concentration (Conidia/mL)	LT_50_	Efficacy (%)	Origin	References
*Beauveria bassiana*						
*B. bassiana*	Laboratory	—	—	80	China	[31]
*B. bassiana* Gxj-10	Laboratory	2.5 × 10^7^	3 d	82.1	China	[33]
*B. bassiana* Xwy-1	Laboratory	2.5 × 10^7^	9 d	60.7	China	[33]
*B. bassiana* Bb1	Laboratory	1 × 10^7^	4 d	95	Philippines	[30]
*B. bassiana* ICIPE275	Laboratory	1 × 10^7^	13 d	80.8	Kenya	[32]
*B. bassiana* 51	Laboratory	1 × 10^7^	16.4 d	77.5	Kenya	[32]
*B. Bassianabassiana* ICIPE56	Laboratory	1 × 10^7^	17.1 d	79.2	Kenya	[32]
*B. bassiana* ICIPE114	Laboratory	1 × 10^7^	12.5 d	84.2	Kenya	[32]
*B. bassiana* R444	Field	10 g/100 L	—	61	South Africa	[22]
*Metarhizium anisopliae*						
*M. anisopliae* Ma1	Laboratory	1 × 10^7^	4 d	100	Philippines	[30]
*M. anisopliae* ICIPE62	Laboratory	1 × 10^7^	9.7 d	89.2	Kenya	[32]
*M. anisopliae* ICIPE21	Laboratory	1 × 10^7^	10.9 d	85	Kenya	[32]
*M. anisopliae* ICIPE7	Laboratory	1 × 10^7^	14.2 d	74.2	Kenya	[32]
*M. anisopliae* ICIPE18	Laboratory	1 × 10^7^	10.9 d	85.8	Kenya	[32]
*M. anisopliae* ICIPE20	Laboratory	1 × 10^7^	13.4 d	80	Kenya	[32]
*M. anisopliae* ICIPE30	Laboratory	1 × 10^7^	12.1 d	75.8	Kenya	[32]
*M. anisopliae* ICIPE45	Laboratory	1 × 10^7^	12.1 d	80	Kenya	[32]
*M. anisopliae* 59	Laboratory	1 × 10^7^	18.5 d	62.5	Kenya	[32]
*M. anisopliae* LBM-30	Laboratory	5 × 10^7^	4.2 d	96.6	Cuba	[34]
*M. anisopliae* LBM-267	Laboratory	5 × 10^7^	4.58 d	100	Cuba	[16]
*M. anisopliae* LBM-5	Laboratory	5 × 10^7^	5.09 d	100	Cuba	[16]
*M. anisopliae* LBM-10	Laboratory	5 × 10^7^	6.91 d	66	Cuba	[16]
*M. anisopliae* LBM-11	Laboratory	5 × 10^7^	6.2 d	96	Cuba	[16]
*M. anisopliae* LBM-12	Laboratory	5 × 10^7^	7.38 d	80	Cuba	[16]
*M. anisopliae* MR	Laboratory	5 × 10^7^	—	62.47	Malaysia	[36]
*bassiana* GHA + M. *brunneum* F52	Field	20 mL/ha + 45 mL/ha	—	100	USA	[39]
*Lecanicillium*						
*Lecanicillium lecanii*	Laboratory	1 × 10^7^	—	74	Indonesia	[39]

### 3.2. Entomopathogenic Bacteria

Among entomopathogenic bacteria used for pest control, *Bacillus thuringiensis* (Bt) is the most widely applied in agriculture [40]. *B. thuringiensis* spores can produce insect-specific toxic effects during their growth [41]. Active Bt insecticidal proteins have been reported in research on the control of *C. formicarius*, including Cry1B, Cry23Aa, Cry37Aa, Cry3Aa, Cry7Aa1 [42,43,44].

Isakova et al. evaluated bioassays on three Cry1B toxin-producing Bt strains (949, 834, 048) [43]. After alkaline dissolution to obtain protoxins and subsequent in vitro activation with trypsin, the proteins were incorporated into artificial diet at 50 mg/mL. While unactivated protoxins showed some insecticidal activity (50% and 17% mortality for strains 949 and 834, respectively), trypsin activation significantly enhanced virulence, resulting in 58–68% larval mortality across all strains. This confirms the high larvicidal potential of activated Cry1B toxins against *C. formicarius* and their promise for development as bioinsecticides. Patricia et al. reported the Cry23Aa and Cry37Aa protein-coding genes respectively from *E. coli* BL21 strain and tested their toxic activity against the 2nd stage larvae of *C. formicarius* [44]. Both proteins were found to be toxic, with Cry23Aa (LC_50_) at 2.12 μg/g and Cry37Aa at 1.25 μg/g. In addition, the study found that the combined administration of hydroxycinnamate and Cry7Aa1 protein increased the mortality rate of *C. formicarius* larvae by more than 10 percentage points compared to Cry7Aa1 alone [41]. By Western blot analysis of the binding of brush border membrane vesicles to Cry3Aa, it was found that the 20 kDa, 30 kDa, 50 kDa and 85 kDa proteins showed immunoaffinity signals with Cry3Aa, among which the 30 kDa protein was identified as similar to annexin IX. The 20 kDa protein is homologous to the heat shock protein Lethal-2, suggesting that these two proteins may act as potential receptors for Cry3Aa toxins and be involved in its toxicity mechanism [45].

Entomopathogenic bacteria are typically the most successful microbial control agents [46], but they are only pathogenic to the larval stages of *C. formicarius*. Moreover, the cryptic endophytic habit of the larvae presents a significant challenge for the effective delivery of bacterial biocontrol agents. This is the main reason why bacterial biocontrol products for this pest are currently relatively limited [11].

### 3.3. Entomopathogenic Nematodes

Entomopathogenic nematodes, (EPNs) have gained increasing attention as effective biological control agents against *C. formicarius* [47,48]. EPNs can actively search for hosts and cause host death within 24 to 48 h through the highly pathogenic symbiotic bacteria they carry, while being safe for humans, animals, non-target natural enemies and the environment [49]. At present, the entomopathogenic nematodes that have been reported for the biological control of *C. formicarius* are mainly Heterorhabditis of the Heterorhabditidae family and Steinernema of the Steinernematidae family [50,51]. Pathogenic effects of entomopathogenic nematodes on the *C. formicarius* (Table 2).

Field trials showed that *H. bacteriophora* HP88 was superior to the chemical insecticides methamidophos and thiazide in reducing the number of *C. formicarius*. The number of *C. formicarius* in the treatment area treated with HP88 nematode suspension was 1.8 to 5.7 times lower than that in the chemical treatment group [50]. *Heterorhabditis* HC1, KM89, OM158, HM108, OM-160 were lethal to both the larvae and pupae of *C. formicarius* and could significantly reduce adult emergence from affected tubers [48,50]. *H. indica* OM-160 reduces the emergence of *C. formicarius* both in the laboratory and in the field [48]. The mortality rate of *S. feltiae* JY-17 against the fifth-stage larvae of *C. formicarius* was 91.67% [52]. Field trials showed that *S. carpocapsae* All was superior to the chemical insecticides methamidophos and thidan in reducing the number of *C. formicarius*. In sweet potato fields treated with All nematodes, the number of *C. formicarius* was 1.8 to 5.7 times lower than that in the chemical treatment group [47]. Measurement of the efficacy of different entomopathogenic nematodes against *C. formicarius*, revealed that the species with stronger pathogenicity against the third-stage larvae of *C. formicarius* were *S. ceratophorum* HQA-87, *S. glaseri* KG and *S. longicaudum* X-7. Adult beetles were generally less sensitive to EPNs [53]. The Hawaiian insect pathogen nematode species *S. feltiae* MG-14 caused a mortality rate of 50% to 60% under indoor conditions and significantly inhibits population occurrence in the field [54].

**Table 2:** Pathogenicity of entomopathogenic nematodes to *C. formicarius*.

Genus	Nematode Species/Strain	Target Worm Stage	Country	Application Method	Application Quantity (“IJs” Infective Juveniles)	Mortality %	References
*Heterorhabditis*	*H. bacteriorphora* North Carolina	Larva	US	Petri dish filter paper method	1668.7 IJs/insect	90	[55]
	*H. megidis*	larvae, pupae, adults	Japan	Petri dish filter paper method	4.75 × 10^5^ IJs/100 mL	80~90	[56]
	*H. bacteriophora* HC1	Adult	Cuba	Petri dish filter paper method	5000 IJs/mL	100	[50]
	*H. indica* KM89	larvae, pupae	US	Culture plate biometry	1–25 IJs/insect	75, 100	[48]
	*H. indica* OM158	larvae, pupae, adults	US	Culture plate biometry	1–25 IJs/insect	92, 92, 9	[48]
	*H.* sp. HM108	larvae, pupae, adults	US	Culture plate biometry	1–25 IJs/insect	92, 92, 8	[48]
	*H. indica* OM160	larvae, pupae, adults	US	Barrel biometric assay	1000 IJs/15 mL	73.2, 56.5, 28.5	[48]
*Steinernema*	*S. carpocapsae* All	Adult	US	Petri dish filter paper method	82.6 IJs/insect	25~60	[55]
	*S. feltiae* MG-14	Adult	US	Petri dish filter paper method	100 IJs/insect	50~60	[54]
	*S. carpocapsae* All	Adult	Japan	Petri dish filter paper method	25,000 IJs/100 mL	90>	[57]
	*S. feltiae*	larvae, pupae, adults	Japan	Petri dish filter paper method	4.25 × 10^3^ IJs/100 mL	70~80	[56]
	*S. feltiae* JY-17	larvae	China	Virulence determination	28 IJs/insect	91.67	[52]
	*S. riobrave*	larvae, pupae	Japan	Petri dish filter paper method	25,000 IJs/100 mL	30~60	[57]
	*S. glaseri* (Mungyeong)	Larva, pupae	Japan	Petri dish filter paper	25,000 IJs/100 mL	30~60	[57]
	*S. glaseri* (Dongrae)	Larva, pupae	Japan	Petri dish filter paper method	25,000 IJs/100 mL	30~60	[57]
	*S. ceratophorum* HQA-87	3 instar, adult	China	Culture plate filter paper biometric assay	500 IJs·cm^−2^	100, 33.3	[53]
	*S. glaseri* KG	3 instar, adult	China	Culture plate filter paper biometric assay	500 IJs·cm^−2^	100, 33.3	[53]
	*S. longicaudum X-7*	3rd instar, adult	China	Culture plate filter paper biometric assay	500 IJs·cm^−2^	100, 33.3	[53]

The combination of pathogenic microorganisms with other techniques can enhance the control effect, such as trapping male insects with sex pheromones and then artificially inoculating and releasing *B. bassiana* to form a “chain of transmission” to expand the control range [58]. Combined application of *B. bassiana* and *M anisopliae* can more effectively reduce tuber damage and increase yield [39]. In addition, plastic mulching combined with five spray applications of the *B. bassiana* TMP1 strain can achieve a control efficiency of 96.76% against *C. formicarius* [51].

Although insect-borne pathogenic microorganisms have considerable potential as biological control agents, the number of registered biocontrol products is limited [39,58] (Table 3). The limited number of commercially registered biological control products is attributable to a combination of interrelated constraints: (1) The substantial costs associated with research and development, comprehensive safety assessments (covering human health, non-target organisms, and environmental impact), field trials, and regulatory registration present a significant economic barrier, often rendering investment returns inadequate for commercialization [59,60]. (2) The efficacy of biological control agents is highly susceptible to environmental variability, resulting in inconsistent field performance, while technical challenges such as the limited shelf life of fungal propagules and difficulties in maintaining the viability of entomopathogenic nematodes further hinder product development and distribution [61,62,63]. (3) In conventional agricultural systems, farmer reliance on rapid-acting chemical pesticides, coupled with lower acceptance of biological alternatives due to their slower mode of action and more demanding application requirements, constrains widespread adoption [19,37].

## 4. Natural Enemies

A few species of known natural enemies of *C. formicarius* (Table 4) were reported. In 1986, wasps *Rhaconotus* sp. and *Bracon* sp. as well as hymenoptera parasitic wasps, were identified on the larvae of *C. formicarius*, but the population was not large enough to suppress the *C. formicarius* population [65]. Jansson and Lecrone reported a hymenopteran parasitoid—*Euderus purpureas*—parasitizing *C. formicarius* in southern Florida yasudai from the south west islands [66]. In the south-west islands of Japan, a new species of *Bracon yasudai* was found and reported with parasitism of 25% of *C. formicarius* in the field [67]. In Cuba and Papua New Guinea, ants were found to be able to effectively control *C. formicarius*, ants were transported from natural areas to fields, rolled up in banana leaves as “temporary nests”, and the rate of *C. formicarius* infestation in areas where ant colonies had accumulated 30 days after planting decreased from 5% to 3% [68,69]. The use of ants as biological agents for the control of *C. formicarius* reduces dependency on chemical pesticides, while concurrently enhancing soil fertility and crop quality. This strategy constitutes a low-cost and sustainable ecological management model, particularly advantageous for resource-constrained smallholder farming systems [70,71,72]. However, the management of *C. formicarius* using parasitoids in the field was not effective due to the primary nocturnal activities of adult *C. formicarius* and the feeding habits of larvae in storage roots [26,71,72,73]. Further screening and large-scale field release of these parasitoids for the control of *C. formicarius* should be pursued, and by reasonably transforming agricultural landscapes, by increasing diverse natural vegetation, the abundance of ants and parasitic wasps can be improved, thereby enhancing the pest control capacity of natural enemies and promoting sustainable agricultural biological control [74].

**Table 4:** Parasitoids for insects of *C. formicarius*.

Family	Genus/Species	Parasitic State	References
Braconidae	*Rhaconotus* sp.	Larva	[65]
Braconidae	*Bracon* sp.	Larva	[65]
Braconidae	*Bracon yasudai*	Larva	[67]
Eulophidae	*Euderus purpureus*	-	[66]
Formicidae	*Pheidole megacephala* (*Fabricius*)	-	[69]

## 5. Botanical Pesticides

Botanical pesticides are a class of green insecticides whose active ingredients are derived from specific parts of plants and are extracted, processed and synthesized with low toxicity, high efficiency, low residue and environmental friendliness [75,76]. Currently, botanical pesticides mainly used to control *C. formicarius* include azadirachtin, tea saponin, pyrethrin, etc. [35,77,78] (Table 5).

Azadirachtin is mainly derived from the neem tree and is one of the most industrialized varieties of plant-based pesticides at present, with high environmental safety [64,79]. In the indoor activity assay, when 1.2% azadirachtin was applied on sweet potato tubers and vine leaves, the mortality rate of adult *C. formicarius* reached 100% within 72–96 h [80]. Field trials showed that when a concentration of 13.95 mg/L of azadirachtin was evenly sprayed on the back of sweet potato leaves and vines, the control effect was 64.92% at 7 days after application, indicating that azadirachtin has a good control effect on adult *C. formicarius*, with a long-lasting effect and relatively stable efficacy [81]. When the growth period and mortality rate of *C. formicarius* under the influence of tea saponin were determined by artificial feed method, the results showed that 1% tea saponin could shorten the larval development period by 4 days, the larval mortality rate by 53.33%, and the adult life by 23.67 days [82]. At a concentration of 2 g/L, the anti-feeding rate of pyrethroids was 97.92%, and the 72 h mortality rate was 78.33% [78]. In addition, 2 g/L of toosendanin had anti-feeding rate as high as 97.87% and a mortality rate of 56.67% [78].

A variety of plant extracts and essential oils have insecticidal potential against *C. formicarius*, such as sesquiterpenoids isolated from *Capraria biflora* L. have been shown to exhibit toxicity against adult *C. formicarius*. The 24 h LC_50_ was 0.902 μg/worm and 1.102 g/worm, respectively [83]. *Hyptis verticillata* Jacq. essential oil had an 80% mortality rate within 48 h at a concentration of 1 μL/g and inhibits 87.20% oviposition and 90% hatching at 4 μL/g [84]. The 48 h LD_50_ of bark oil from *Bursera hollickii* (Britton) was 12 μg/g [85]. Liu et al. identified five antenna-active volatile compounds from sweet potatoes infested by *C. formicarius* larvae [13]. Among these, farnesol, citronellol, nerol, and geraniol significantly repelled female weevils from feeding and oviposition, laying the groundwork for developing repellents or oviposition inhibitors. In addition, the leaf crude extract protein of the wild *Ipomoea mauritiana* had a 48 h mortality rate of 40–50% at concentrations of 4–5 mg/mL [86].

Although botanical pesticides have achieved some success in controlling *C. formicarius*, many current studies on the effects of plant extracts and essential oils have been limited to the laboratory stage and have not fully demonstrated their effectiveness in practical applications [11,83,86]. In the future, efforts should be made to increase research on the fundamentals of the application to move this type of agent from experimental research to field practice, so as to achieve large-scale and standardized application in field production.

**Table 5:** Reported plant-based pesticides for *C. formicarius*.

Species	Active Ingredients	Application Concentration	Experimental Conditions	Efficacy (%)	References
*Azadirachta indica* A. Juss	Azadirachtin	1.2%	Laboratory	100% mortality	[80]
*Azadirachta indica* A. Juss	Azadirachtin	13.95 mg/L	Field	64.92% control effect	[81]
*Camellia sinensis* (L.)	Tea saponin	1%	Laboratory	53.33% mortality	[82]
	Matrine	1.3%	Field	82.64% control effect	[81]
	Rotenone	6%	Field	65.40% control effect	[81]
*Tanacetum cinerariifolium* (Trevir.)	25% pyrethroids	2 g/L	Laboratory	78.33% mortality	[78]
*Melia toosendan* Sieb. et Zucc.	2% toosendanin	2 g/L	Laboratory	56.67% mortality	[78]
*Croton linearis* Jacq.	Diterpenoids	0.32 μg/insect	Laboratory	50% mortality	[87]
*Manihot esculenta* Crantz	Petroleum ethers	5.0%	Laboratory	86.7% mortality	[88]
*Capraria biflora* L.	Sesquiterpenoids	0.902 mg/insect	Laboratory	50% mortality	[83]
*Hyptis verticillata* Jacq.	Sesquiterpenoids	60 mg/insect	Laboratory	80–100% mortality	[83]
*Hyptis verticillata* Jacq.	Sesquiterpenoids	1 μg/insect	Laboratory	80% mortality	[84]
*Bursera hollickii* (Britton)	Bursera	12 μg/g	Laboratory	50% mortality	[85]
*Cleome viscosa* L.	Pyrethroids	3 µg/insect	Laboratory	30% mortality	[89]
*Nicotiana tabacum* L.	Chloroform and acetone extracts	20 µg/insect	Laboratory	100% mortality	[90]
*Ipomoea mauritiana*	Crude protein extract	4–5 mg/mL	Laboratory	40–50% mortality	[86]

## 6. Insect Sex Pheromones

Insect sex pheromones are volatile chemical substances secreted outside the body by the sex pheromone glands of insects, which can be perceived by individuals of the opposite sex and induce behaviors and physiological responses such as mate seeking, directed courtship, and mating [91]. Sex pheromone technology has been widely applied in pest monitoring, mating interference, pest quarantine and mass trapping due to its trace amount, high efficiency, high sensitivity and good compatibility with non-target biosafety and the environment [92,93].

The sex pheromone component of *C. formicarius* (Z)-3-dodecen1-ol (E)-2-butenoate was first extracted and separated using n-hexane and liquid chromatography in 1978 [94] and was artificially synthesized in 1986. Field trials demonstrated its strong luring activity on male adult *C. formicarius* [95]. Sex pheromone traps are widely used as mass trapping in field control due to their high efficiency and large trapping capacity (Table 6). Pillai et al. found that placing one sex pheromone trap per 100 m^2^ could effectively reduce the population of *C. formicarius* from 39% to 9.5% respectively, while increasing sweet potato yield by 53% [96]. Similarly, when traps were placed at intervals of 15 m, the damage rate of tubers was reduced by 8.5% to 10.1%, and the control effect was 53.1% to 58.2% [97]. Reddy et al. found that barrel sex pheromone traps could effectively reduce damage to sweet potatoes [98]. Dilipkumar et al. developed a simple, low-cost, efficient plastic rod trap that has been promoted in Malaysia for population monitoring and mass-trapping efforts [99]. In addition, sex pheromone is more effective when combined with other control methods. For example, the trapping ability of sex pheromones can be significantly enhanced by adding light sources of different colors. When combined with green light sources, the number of male insects can be increased fivefold [100]. Therefore, in practical application, it is recommended to use efficient and low-cost types of traps, and to focus on coordinating with other control methods such as fungicides to build an integrated control system.

The reception and perception of sex pheromones are inseparable from olfaction, which plays a crucial role in the behaviors of insects, including host plant recognition, choosing mates, spawning sites and escaping predators [101]. Odorants enter through the olfactory receptor pores and are transported by odorant binding proteins (OBPs) across the receptor lymph fluid. Upon reaching the olfactory receptor neurons, they initiate a cascade of chemoelectrical transduction [102,103]. Olfactory receptors convert these external chemical stimuli into electrical signals, which are then propagated along axons to the central nervous system. Once there, the signals are integrated and processed, ultimately leading to the generation of specific behavioral responses [91,104]. OBPs, as an important component of olfactory recognition, can be divided into sex pheromone-binding proteins and common odor-binding proteins, which are respectively involved in the recognition of sex pheromones and plant volatiles [105,106,107]. A total of 36 OBPs (CforOBP1–36) have been identified in *C. formicarius*. Jia et al. found that CforOBP8 has the strongest binding ability to sex pheromones (Z)-3-dodecen1-ol (E)-2-butenoate and can also bind to a variety of plant volatiles [108]. Hua et al. demonstrated that CforOBP1–3 has a high affinity for sex pheromones, and that CforOBP1 can also bind to a variety of plant secondary metabolites [109]. After RNA interference silencing of these genes, the behavioral responses of insects to sex pheromones and host plants were significantly weakened. Further studies showed that CforOBP4–6 also had a strong binding force to sex pheromones, and that CforOBP5 and CforOBP6 specifically recognized volatile glycosides and pigment substances in sweet potatoes. In addition, plant volatiles such as limonene can significantly induce the antennae potential and tendency behavior response of *C. formicarius* [108]. These findings suggest that sex pheromones and host volatiles can act as attractants or enhancers to interfere with host localization and egg-laying behavior of pests, providing a theoretical basis for the development of novel control tactics [96,109,110,111].

Research on sex pheromone trapping techniques had achieved certain results in places such as China and Malaysia [96,98], and studies on their olfactory recognition mechanisms continue to advance [108,109,110]. However, there are still problems such as insufficient research on the synergistic mechanism of decoders in field applications, which restrict its further promotion and improvement of effect.

**Table 6:** Sex pheromone trapping effect on *C. formicarius*.

Pheromone Component	Concentration	Trap Type	Trap Densitie	Intervals	Trapping Effect (%)	References
(Z)-3-dodecen1-ol (E)-2-butenoate	1 mg	-	1 trap/100 m^2^	10 m	40~70	[96]
(Z)-3-dodecen1-ol (E)-2-butenoate	-	-	2 traps/667 m^2^	15 m	53.1~58.2	[97]
(Z)-3-dodecen1-ol (E)-2-butenoate	10 mg	Pherocon Unitrap	-	10 m	50	[98]
(Z)-3-dodecen1-ol (E)-2-butenoate	0.1 mg	Plastic Pole Trap	-	10 m	60~78	[99]
(Z)-3-dodecen1-ol (E)-2-butenoate	12 mg	Funnel-type Solar Green LED Trap	-	5 m	204	[100]

## 7. Transgenics

Bt insecticidal protein transgenic plants play a significant role in pest management [88,112]. In 1998, Moran et al. modified the CryIIIA gene by adding promoters and enhancers and introduced it into sweet potato (*I. batatas* cv. “Jewel”), successfully obtaining transgenic plants resistant to *C. formicarius* [113]. Subsequently, Garcia et al. transformed the stems and leaves of sweet potatoes with *Agrobacterium tumefaciens* carrying nptII and *B. thuringiensis* endotoxin genes and found that weevil damage on tubers was up to 5 times lower in transgenic compared with control plants [114]. Ekobu et al. evenly mixed seven Bt Cry proteins into the feed to determine their toxicity, among which the LC_50_ of Cry7Aa1, ET33/34 and Cry3Ca1 proteins were less than 1 μg/g, providing candidate protein genes for breeding transgenic insect-resistant sweet potato varieties [115]. After the Cry8db gene was introduced through transgenic technology, with the increase in transgenic expression level, the degree of damage to the tubers to *C. formicarius* was significantly reduced [116]. The cry3Ca1 gene was introduced into Kb1 sweet potatoes by *Agrobacterium* conversion, and it was found that the root pest rate of transgenic lines was 1.31–1.35 times lower than that of the non-transgenic control, and the overall pest rate was 7.5–16.6% lower [117]. Bt transgenic sweet potatoes can significantly enhance yield potential while improving resistance to pests and diseases. Moreover, decreased reliance on chemical pesticides further lowers production costs, thereby increasing farmers’ income and improving their livelihoods [114,115,116,117]. Although significant progress has been made in developing insect-resistant germplasm in Bt transgenic sweet potatoes, the expression levels of effective insecticidal protein toxins in the tubers are very low and have no significant impact on adult mortality [11,118]. The approval process for transgenic crops is complex and time-consuming, and public acceptance is limited. Some countries even prohibit the cultivation of transgenic sweet potatoes, which hinders their market promotion [119]. In addition, the potential risks of foreign genes still require long-term monitoring, further increasing research and development costs and timelines. Therefore, transgenic sweet potatoes are still in the early stages [11,118].

## 8. RNA Interference (RNAi) Technology

RNA interference, an effective gene silencing mechanism in eukaryotes [120], shows great potential in pest management strategies and is an innovative biological control strategy [121,122]. In recent years, RNAi techniques have been applied to control *C. formicarius* by designing dsRNAs targeting essential genes [123,124,125].

Prentice et al. found 47 RNAi-related genes in the second instar larvae of *C. formicarius* and selected the laccase2 dsRNA (length 362 bp, dose 0.2 μg/mg) to be injected into the blood cavity of the second instar larvae, with dsRNA of the gfp gene as the control [123]. The results showed that the laccase2 mRNA level decreased by 91.7% after 24 h compared with the control. Phenotype showed untanned cuticle in the larval head after 3 days, no hardening of the keratin structure in the pupal stage after 5–6 days, complete inhibition of hardening of the cuticle layer of the adult skeleton, soft, deformed and no pigmentation of the body wall, ultimately leading to death, indicating a systemic RNAi effect. Christiaens injected 24 target-gene dsRNAs into second-instage larvae (0.2 μg/mg/head), while the control group was injected with ds*GFP*. After 14 days, all tested dsRNAs except for those targeting Syb, Pfk, Mad1, and rpl135 genes had a mortality rate of over 60%. This mortality rate was much higher than 24% in the control group. Further, better-performing dsRNAs targeting vha68-2, adk2, prosα2, rps13 and snf75 were selected for oral bioassays, with a mortality rate of 95% [124]. By RNA interference silencing of CforOBP1-3 and CforCSP1/5/6, it was found that CforOBP1-3 defective *C. formicarius* partially lost their sense of smell, and their ability to locate pheromones and sweet potato volatiles decreased. The CforCSP1/5/6 defective *C. formicarius* has a reduced ability to locate sweet potato volatiles [110,126]. The silencing efficiency of dsRNA on gene wupA was evaluated by injection and oral feeding of different ages of *C. formicarius*. After 10 days of injection of ds*wupA* (250 ng/μL), the mortality rates of 1st, 3rd, and 5th instar larvae were 90%, 85%, and 70%, respectively, while it also showed significant growth inhibition on the 1st instar larvae [125]. The combination of RNAi with other pest management methods yielded better results [127,128]. RNAi technology has transitioned from a conventional “broad-spectrum” approach to a more targeted “precision regulation” strategy [120,121,122]. This shift holds promise for delaying the development of pest resistance, reducing reliance on frequent pesticide applications, and ultimately lowering agricultural production costs [121]. However, the design, synthesis, and large-scale production of dsRNA remain costly, and the development of effective formulations and delivery systems requires substantial research, development, and financial investment. These high initial costs are likely to be transferred to the end product, potentially placing it out of reach for small-scale farmers. Consequently, the adoption and widespread application of this technology among a broad farmer population may be significantly constrained [129,130]. Moreover, RNA biopesticides have limitations such as precise delivery, low persistence, and poor stability [131,132]. At present, the registration of RNA biopesticides is still in its infancy, with only a few products entering the market [133], and there are still some problems that need to be urgently solved for the industrial application of RNA pesticides [134]. First, in terms of pesticide product registration, registration and regulating authorities should actively promote the formulation of standards for pesticide product registration, strengthen the formulation of regulatory policies, and enhance the safety assessment of RNA pesticides to ensure the safety of non-target organisms and the ecological environment [59,60]; Secondly, processes such as fermentation and separation purification to meet industrialization and marketization demands should be optimized [135]; Finally, it is essential to develop new strategies for sustainable, multi-host-compatible protection pest control based on RNAi, such as using host-associated probiotics to express dsRNA targeting insects [136].

## 9. Breeding of Resistant Varieties and Resistance Mechanisms

Developing and planting resistant varieties is a key approach to controlling *C. formicarius*, reducing the use of chemical insecticides, lowering production costs, and promoting green and sustainable development of the sweet potato industry [137,138]. Therefore, screening and identifying resistant sweet potato varieties are at the core of breeding for resistance to *C. formicarius* [40,139].

### 9.1. Existing Resistant Varieties

Based on field and indoor evaluations, a classification system has been established with indicators such as hazard index, insect-infested rate, and number of feeding holes as the core to classify the resistance of sweet potato varieties to *C. formicarius* into five grades: high resistance, medium resistance, resistance, susceptibility, and high susceptibility [140,141,142] (Table 7). Despite the successive screening of multiple resistant sweet potato germplasm resources worldwide, there has been limited progress in developing varieties with both high yield and stable pest resistance over the years [143,144]; in addition, there is a lack of uniform resistance identification standards in various countries, and the identified resistant varieties cannot be promoted and applied. An international standard for the identification of resistance to *C. formicarius* needs to be established [145,146,147,148]. Currently, the main bottleneck hindering the widespread promotion and application of sweet potato weevil-resistant varieties lies in their overall agronomic performance: on one hand, the yield levels of some resistant varieties are not competitive; on the other hand, their tubers often have low starch and sucrose content, directly affecting taste and processing quality [141,149].

### 9.2. Advances in Resistance Mechanisms

#### 9.2.1. Morphological and Physical Resistance

The morphological characteristics of sweet potato tubers and the depth of tuber formation are important agronomic traits that affect their resistance to *C. formicarius* [88]. Round and oval sweet potato tubers are more vulnerable to *C. formicarius* than long-stemmed, spindle-shaped and slender ones [153]. Deep-rooted early-maturing varieties are significantly less affected than shallow-rooted late-maturing varieties by up to four times, mainly because the deep-rooted structure reduces tuber exposure in the soil [7]. Path analysis shows that the length of the tuberous neck has a significant direct effect on tuberous damage caused by *C. formicarius* and can be used as a reliable predictor in resistance breeding [154]. In addition, the number of adults in the tubers was negatively correlated with the dry matter content of sweet potatoes, suggesting that the dry matter content of tubers is an effective indicator for screening resistant varieties [141].

#### 9.2.2. Secondary Metabolites

Chemicals in sweet potatoes play a key role in their resistance mechanism to *C. formicarius* [143]. Earlier studies found that sweet potato varieties rich in beta-carotene were more vulnerable to damage [149]. In laboratory bioanalysis of the food and egg-laying preferences of *C. formicarius* to four varieties (Centennial, Jewel, Resisto, Regal), combined with gas chromatography analysis, it was found that the variety Centennial was most favored by females, and its surface contained a specific triterpenol acetate (later identified as bodisterone acetate). Further studies confirmed that the compound significantly stimulated oviposition at doses as low as 0.04 μg in a dose-dependent manner, but did not affect feeding behavior, indicating its specificity as an oviposition stimulant and as a chemical marker in resistance breeding [155,156,157,158,159]. Korada et al. identified cyclopropane fatty acid esters using a double selection olfactometer as diagnostic markers for rapid screening of resistant varieties [160]. In addition, the tuberous bark and latex of the New Kawogo variety are rich in hydroxycinnamate, which has a significant dose-dependent toxic effect on larvae [42,161]. The leaves of the sweet potato Tainong57 release a large amount of (E)-4,8-dimethyl–1,3,7-nonatriene (DMNT) after being affected, which activates the activity of the protease inhibitor sporamin in adjacent plants and enhances the resistance of sweet potatoes to *C. formicarius* [162]. The study found that exogenous chlorogenic acid treatment significantly mitigated the feeding damage to leaves caused by *C. formicarius* [163]. Correlation analysis further revealed that starch and sucrose content in the tubers were significantly positively correlated with the number of pests and the rate of tuber damage, suggesting that low sugar characteristics could be used for indirect screening of resistant varieties [141].

#### 9.2.3. Molecular Mechanisms

Yada et al. used SSR to conduct genetic diversity analysis on the sweet potato two-parent hybrid population (insect-resistant New Kawogo × insect-sensitive Beauregard) and screened out 133 highly polymorphic markers, laying a material foundation for subsequent screening of insect-resistant genes [164]. Further studies identified that markers IBS11, IbE5 and IbJ544b were significantly associated with resistance and hydroxycinnamate content and could serve as potential auxiliary markers [165]. Genome-wide association analyzes were conducted to detect resistance-related regions in the Ib04-5 linkage group of the 90IDN-47 variety and the Ib05-1 linkage group of the PSL variety [166]. Two resistant QTLs were identified by SNP mapping, located in linkage groups 14 and 3 [167]. The resistant variety Kyushu No.166 upregulated multiple terpene synthesis genes itf09g05600.t1, itf09g05580.t1, and itf12g13950.t1 after infection, enhancing resistance by disrupting the larval pupal process [142]. Jasmonic acid, salicylic acid and abscisic acid increased after infection and enhanced defense by regulating the expression of key genes for chlorogenic acid synthesis (IbPAL, IbC4H and IbHQT) [163]. Xiao et al. further revealed by GC-MS, stable isotope tracing, transcriptional and metabolomics that sweet potatoes rhythmically released (Z)-3-hexenyl acetate and allo-ocimene after injury, which were synthesized through the accumulation of injury substrate (Z)-3-hexenol and JA-mediated upregulation of the IbOS gene, respectively [168]. And it can act as a gaseous signal to activate the defense response of neighboring plants. The team of Hou Xingliang used the resistant × susceptible hybrid population for QTL mapping, identified two major insect-resistant gene loci, and cloned the key insect-resistant genes SPWR1 (WRKY transcription factor) and SPWR2 (quinic acid synthase gene) for the first time, revealing that they enhance insect-resistance by activating the quinic acid metabolic pathway. This provides important targets for molecular breeding of insect-resistant sweet potatoes [151].

## 10. Conclusions and Future Perspectives

*C. formicarius* is a devastating pest that harms the sweet potato industry. Its larvae bore into the tubers, causing rot, and the adults feed on the stems and leaves, seriously affecting the yield and quality of sweet potatoes [8,10,11]. This pest has a covert nature and is difficult to control effectively through chemical control, and the long-term use of chemical pesticides can also cause problems such as pesticide resistance, environmental pollution and agricultural product safety risks [19,20,21,22]. Therefore, the development of biological and other pest management techniques and the establishment of a green integrated control system have become an inevitable trend in the control of *C. formicarius*.

### 10.1. Most Promising Strategies and Bottlenecks

Among the various approaches, entomopathogenic fungi and sex pheromone-based trapping currently show the most immediate promise for field deployment, while the widespread adoption of transgenic and RNAi-based solutions faces significant regulatory and public acceptance bottlenecks [39,96,99,129,130]. The most promising management strategies can be prioritized into distinct readiness categories based on their current development stage, advantages, and key bottlenecks. Entomopathogenic fungi, such as *B. bassiana* and *M. anisopliae*, alongside sex pheromone-based trapping, represent near-term field-ready options [39,58,100]. Entomopathogenic fungi demonstrate high pathogenicity, reducing feeding, reproduction, and field damage when properly formulated, though their efficacy is constrained by sensitivity to UV radiation, heat, and desiccation. Pheromone traps offer species-specific, non-toxic monitoring and mass-trapping potential, but require optimization in deployment density, synergists (e.g., host volatiles), and area-wide coordination to improve cost-effectiveness [61,96,98,108].

In the mid-term, botanical pesticides and natural enemies show promise but need stronger field validation and standardization [73,74,83]. Plant-derived repellents and essential oils provide lower residue risks and potential behavioral disruption yet lack consistent field persistence and scalable formulations [83,84]. Conservation biocontrol using parasitoids or predatory ants can offer sustainable suppression but is limited by insufficient mass-rearing systems and landscape-dependent efficacy [70,71,72,73].

For the long-term, host plant resistance forms the most durable foundation, with accelerated progress through QTL mapping and gene cloning [137,138,151]. Complementary biotechnological approaches—such as Bt transgenics and RNAi exhibit high specificity and potential but face significant barriers, including regulatory hurdles, public acceptance, and challenges in delivery, stability, and field persistence [114,129,130].

### 10.2. Integrated Pest Management Framework for C. formicarius

(1) Early to Mid-Season: Monitoring and Threshold-Based Intervention

A pheromone-based trapping system serves as the cornerstone for monitoring, providing early detection of adult activity and tracking population trends [92,93]. When trap captures indicate a rising population, mass trapping should be deployed [99]. Applications of entomopathogenic fungi or entomopathogenic nematodes can then be timed to coincide with peaks in adult movement, maximizing exposure and efficacy [19,51].

(2) Population Suppression: Integration of Behavioral and Biological Controls

Mass trapping functions both to lower adult densities and disrupt mating [93]. Concurrently, ENF and EPN applications exert direct biological mortality [19,51]. such as “attract-and-infect” where pheromones lure pests to pathogen-treated stations that can amplify control by facilitating secondary transmission [58]. Once ecological stability is achieved, botanical insecticides or repellents may be introduced as supplementary measures, for example, to deter oviposition or protect susceptible plant stages [77,78].

(3) Ecological Reinforcement: Fostering a Resilient Agroecosystem Long-Term

By diversifying habitats and implementing strategic landscape management, populations of natural enemy species can be conserved and enhanced, thereby effectively improving the overall sustainability of the system [74].

(4) Prospective Enhancements: Toward Next-Generation Precision Control

Pending regulatory approval and viable delivery systems, RNA interference (RNAi)-based targeting could be incorporated, potentially in combination with pheromone-guided deployment [59,60,135]. Similarly, the adoption of Bt-expressing or gene-edited host-plant resistance should be accompanied by structured resistance-management plans to ensure durability [116,136].

### 10.3. Future Perspectives

Currently, the green control system for *C. formicarius* still has several key research gaps that urgently need to be addressed. Regarding entomopathogenic fungi, efforts should focus on developing formulations with strong stress resistance and long field persistence [61,62]. Research on plant-derived pesticides needs to move from the laboratory to the field, systematically verifying their field effectiveness and developing new formulations that are long-lasting and stable [83,86]. The use of natural enemy insects should enhance resource screening, large-scale breeding, and field release techniques [67,74,169,170]. Resistance breeding requires an in-depth analysis of genes related to pest resistance and their metabolic pathways [151,168]. RNA interference technology needs to overcome technical bottlenecks in dsRNA delivery efficiency and field stability, while strengthening target gene screening and non-target safety evaluation [60,135,136]. In addition, the promotion of genetically modified insect-resistant sweet potatoes still requires the further improvement of safety evaluation systems and enhanced scientific communication to facilitate regulatory development and social acceptance [118,119].

## Figures and Tables

**Table 3:** Registered biocontrol products in controlling *C. formicarius*.

Product Name	Active Components	Registration Regions	Application Parameter	References
EcoBb	*B. bassiana* R444	South Africa	10 g/100 L	[19]
Botanigard	*B. bassiana* GHA	USA	2.14 kg/ha	[37]
Met Maste	*M. anisopliae*	USA	-	[37]
Aza-Direct	1.2% Azadirachtin	USA	10 mL/L of water	[64]
Conserve SC	11.6% Spinosyn	USA	0.5 mL/L of water	[64]

**Table 7:** Resistance of sweet potato varieties to *C. formicarius*.

Varieties	Experimental Conditions	^a^ Resistance Level	Country	References
Pu Shu 32	Field, Laboratory	MR	China	[140]
E Shu 15	Laboratory	HR	China	[150]
N73	_	HR	China	[151]
N28	_	HR	China	[151]
Saribu Dolok Simalungun	Field	R	Indonesia	[148]
UNC2016. CIL/JPV-04	Laboratory	MR	Indonesia	[142]
UNC2016. CIL/JPV-05	Laboratory	MR	Indonesia	[142]
Beta2	Laboratory	MR	Indonesia	[142]
Kidal	Laboratory	MR	Indonesia	[142]
New Kawogo	Field	MR	Uganda	[146]
RAK865 (Dimbuka)	Field	MR	Uganda	[146]
HMA519 (Kyebagambire)	Field	MR	Uganda	[146]
LIR302 (Anamoyito)	Field	MR	Uganda	[146]
Obugi	Field	HR	Kenya	[141]
5Nyandere	Field	HR	Kenya	[141]
Mogesi Gikenja	Field	MR	Kenya	[141]
Bungoma	Field	MR	Kenya	[141]
292-H-12	Field	MR	Kenya	[141]
Santo Amaro	Field	MR	Kenya	[141]
9 Nduma	Field	MR	Kenya	[141]
Kenspot 3	Field	MR	Kenya	[141]
Wera	Field	MR	Kenya	[141]
1-Ujili	Field	MR	Kenya	[141]
Mugande	Field	MR	Kenya	[141]
Kenspot 2	Field	MR	Kenya	[141]
Murasaki	Laboratory	HR	America	[147]
BSP-1	Field	MR	India	[145]
BSP-22	Field	MR	India	[145]
BSP-26	Field	MR	India	[145]
BSP-27	Field	MR	India	[145]
BSP-32	Field	MR	India	[145]
CHFSP-10	Field	R	India	[3]
CHFSP-14	Field	R	India	[3]
CHFSP-15	Field	R	India	[3]
*Ipomoea mauritiana*	Laboratory	R	India	[152]

^a^ level of resistance of sweet potato varieties to *C. formicarius*. HR, Highly Resistant; MR, moderately resistant; and R, resistant.

## Data Availability

No new data were created or analyzed in this study. Data sharing is not applicable to this article.
